# Mass spectrometry-based peripheral blood proteomics for biomarker discovery in idiopathic pulmonary fibrosis

**DOI:** 10.1186/s12931-025-03377-5

**Published:** 2025-10-22

**Authors:** Aravind A. Menon, Benedikt Gansen, Hillary Mulder, Megan L. Neely, Panagiotis Papavasileiou, Margaret L. Salisbury, Brian D. Southern, Christian Hesslinger, Thomas B. Leonard, Felix Meissner, Jamie L. Todd

**Affiliations:** 1 Medical University of South Carolina, Charleston, SC USA; 2 University Hospital Bonn, Bonn, Germany; 3 Max Planck Institute of Biochemistry, Martinsried, Munich, Germany; 4 Duke Clinical Research Institute, Durham, NC USA; 5 Duke University Medical Center, Durham, NC USA; 6 Boehringer Ingelheim Pharma GmbH & Co. KG, Biberach, Germany; 7 Vanderbilt University Medical Center, Nashville, TN USA; 8 Cleveland Clinic, Cleveland, OH USA; 9 Boehringer Ingelheim Pharmaceuticals, Inc., Ridgefield, CO USA

**Keywords:** Interstitial lung diseases, Observational study, Proteome, Registries

## Abstract

**Background:**

The circulating proteome may provide insights into the pathobiology of idiopathic pulmonary fibrosis (IPF) and diagnostic or prognostic biomarkers. We applied liquid chromatography coupled to mass spectrometry to quantify the peripheral blood proteome in patients with IPF and identify proteins associated with disease severity and progression.

**Methods:**

The IPF cohort comprised 299 patients from the IPF-PRO Registry. Controls (*n* = 99) without known lung disease had similar distributions of age, sex and smoking status to the IPF cohort. Proteins were measured in plasma collected at enrollment using an Evosep One coupled to an Orbitrap Exploris. Data were analyzed with Spectronaut 14 with a deep experimental spectral library and were log_2_ transformed. Linear regression was used to compare protein abundances in the IPF versus control cohorts and identify proteins associated with disease severity measures at enrollment in the IPF cohort. Cox regression analyses were used to identify proteins associated with outcomes in the IPF cohort, split 75/25 into training and test sets. The false discovery rate was controlled at 5%.

**Results:**

Overall, 761 protein groups corresponding to 736 unique genes were detected. Of these, 168 protein groups were significantly different in abundance in the IPF versus control cohorts, of which 39 were ≥ 1.3-fold different. Among the top differentially expressed proteins were surfactant protein B (SFTPB), secretoglobin family 3A member 1, intercellular adhesion molecule 1, thrombospondin 1 and platelet factor 4. In patients with IPF, greater abundance of apolipoprotein A-1 was statistically significantly associated with higher forced vital capacity % predicted at enrollment, while greater abundance of fibulin-1 was statistically significantly associated with lower diffusing capacity of the lungs for carbon monoxide % predicted. Multivariable models selected 4 proteins (SERPINA7, SFTPB, alpha 2 HS glycoprotein, kininogen 1) and 3 clinical factors that best discriminated the risk of respiratory death or lung transplant in patients with IPF, with a C-index of 0.78 in the training set and 0.72 in the test set.

**Conclusions:**

Mass spectrometry-based proteomic analysis of data from the IPF-PRO Registry confirmed proteins previously associated with the presence, severity and progression of IPF and revealed new candidate biomarkers.

**Trial registration:**

ClinicalTrials.gov; No: NCT01915511; registered August 5, 2013; URL: www.clinicaltrials.gov.

**Supplementary Information:**

The online version contains supplementary material available at 10.1186/s12931-025-03377-5.

## Background

Idiopathic pulmonary fibrosis (IPF) is a progressive fibrotic lung disease associated with high mortality [1]. Despite advances in radiological and serological testing, it remains a disease with significant diagnostic and prognostic challenges [2, 3]. Patients with IPF show heterogeneous disease trajectories [4, 5]. The development of biomarkers to aid in prognostic assessment remains an unmet need. Several circulating protein biomarkers have shown promise as predictors of disease progression or mortality [6–10], but none is accurate enough to justify its use in clinical practice.

Quantifying the circulating proteome in patients with IPF may provide insights into the pathobiology of this disease and uncover candidate diagnostic or prognostic biomarkers. Proteomic analyses in IPF have typically employed approaches that are limited to target primers, such as aptamer-based technologies or proximity extension assays [8, 10, 11], which do not directly quantify proteins or allow discovery of biomarkers beyond a predefined list of targets. Mass spectrometry-based proteomics represents an orthogonal and complementary methodology that offers distinct advantages, specifically the unbiased detection of abundant disease-associated proteins not covered on targeted panels. In this study, we applied liquid chromatography (LC) coupled to mass spectrometry (MS) to quantify the peripheral blood proteome in patients with IPF and controls of similar age and sex distribution. Further, in the IPF cohort, we identified proteins associated with disease severity and progression during follow-up.

## Methods

### Cohorts

The IPF cohort comprised patients enrolled in the Idiopathic Pulmonary Fibrosis Prospective Outcomes (IPF-PRO) Registry (ClinicalTrials.gov; No: NCT01915511; registered August 5, 2013) at US sites between June 2014 and February 2017 [12]. Patients had IPF that was diagnosed or confirmed at the enrolling center in the past six months, according to the 2011 international diagnostic guidelines [13]. A blood sample and demographic/clinical data were collected at enrollment. Following enrollment, clinical data were collected as part of patients’ routine care until death, lung transplant, or withdrawal from the registry. Outcomes were ascertained from enrollment through March 2023. Deaths were reported as due to a respiratory cause or not due to a respiratory cause by the investigator. Patients in this analysis had an enrollment blood sample that passed quality control measures for proteomics.

The control cohort was drawn from the Measurement to Understand the Reclassification of Disease of Cabarrus/Kannapolis (MURDOCK) Study Community Registry and Biorepository, a longitudinal cohort study of adults in North Carolina (NCT0170840) [14]. Participants were white; non-Hispanic; aged 60 to 80 years; had no self-reported respiratory disease, cancer, or autoimmune disease; did not smoke or have second-hand tobacco exposure; did not use respiratory-targeted medication or immunomodulators. Random sampling, stratified by age, sex and smoking status (ever/never), was used to select 100 controls with a similar distribution of these characteristics to the IPF cohort.

### Sample preparation

Plasma samples from the two cohorts were processed together, with samples randomized by patient to mitigate batch effects. Commercial reference samples were analyzed in parallel to evaluate workflow performance. Samples were processed with an automated liquid handling platform (Agilent Bravo). Plasma samples were not immunodepleted prior to MS protein detection and quantification. However, to increase protein coverage and boost peptide identification during downstream data processing, a deep experimental spectral library was generated from100 random IPF and control samples that were pooled and depleted of the seven most abundant plasma proteins (albumin, immunoglobulin A, immunoglobulin G, transferrin, haptoglobin, antitrypsin and fibrinogen). Proteins were then reduced, alkylated and digested. Two additional experimental spectral libraries were created from pools of peptides taken from digested samples, containing exclusively IPF or exclusively control samples. The peptide pools were fractionated. Details are provided in Additional file 1, section S1.

### LC–MS/MS

Peptides were analyzed by an Evosep One (Evosep) LC [15] coupled to an Exploris Orbitrap 480 MS (Thermo Scientific) by a nanoelectronspray ion source [16]. Peptide identification and spectral matching from data-dependent MS raw files of the experimental spectral library samples were performed with the computational proteomics platform MaxQuant [17] version 1.6.14. Protein inference was performed using the Uniprot reference proteome. An experimental spectral library was generated from the MaxQuant output files and the corresponding MS raw files using Spectronaut (Biognosis) version 14. Protein identification and quantification from data-independent MS raw files of the cohort samples were performed with Spectronaut. Spectral matching and peptide identification were performed using the deep experimental spectral library, which improved both depth and coverage (see Additional file 1: Fig. S1). Label-free quantification was performed with QUANT 2.0. The results were log_2_ transformed and missing values were imputed by downshift imputation [18]. Batch correction was performed using pyComBat. Principal component analysis (PCA) after batch correction showed good clustering of the reference samples (see Additional file 1: Fig. S2). Details are provided in Additional file 1, section S1.

### Statistical analyses

Linear regression was used to compare protein abundances (dependent variable) in the IPF vs. control cohorts (independent variable). Comparisons between specific antifibrotic treatment groups (nintedanib, pirfenidone, no treatment) and controls were performed to determine if differences in protein abundance between controls and patients with IPF varied by antifibrotic drug use. Additionally, among patients with IPF, protein concentrations were compared between subsets based on antifibrotic drug use (nintedanib versus no treatment, pirfenidone versus no treatment, nintedanib versus pirfenidone). *P*-values were corrected for multiple comparisons using the Benjamini–Hochberg procedure to control the false discovery rate (FDR) at 5%. Proteins were regarded as significantly different in abundance between groups if FDR-corrected *p* ≤ 0.05. Enriched pathways in the IPF versus control cohorts were detected by performing over-representation analysis on the gene level with clusterProfiler [19]. The canonical pathways set from Reactome [20] was used as a reference set. The background set was defined by all genes corresponding to all detected protein groups. Pathways with FDR-corrected *p* < 0.01 were considered significantly enriched.

Among patients with IPF, linear regression was used to determine proteins associated with disease severity measures at enrollment (forced vital capacity [FVC] % predicted and diffusing capacity of the lungs for carbon monoxide [DL_CO_] % predicted). In these models, protein concentration was considered the independent variable and disease severity measure the dependent variable. Proteins were regarded as statistically significantly associated with a disease severity measure if FDR-corrected *p* ≤ 0.05 and clinically significantly associated if there was a ≥ 5-unit difference in disease severity measure per twice the protein concentration. Analyses were unadjusted and adjusted for antifibrotic therapy at enrollment. There was no relationship between smoking status and FVC % predicted at enrollment, but smoking status was significantly associated with DL_CO_ % predicted at enrollment. Thus, the analyses for DL_CO_ % predicted % predicted were additionally adjusted for smoking status (ever vs. never) at enrollment.

Univariable Cox regression analyses were used to identify proteins associated with time to 1) composite of absolute decline in FVC % predicted ≥ 10%, death, or lung transplant and 2) composite of respiratory death or lung transplant. In the analysis of the latter composite, non-respiratory deaths were censored as non-events. Analyses were unadjusted and adjusted for clinical covariates (sex, age, FVC % predicted, DL_CO_ % predicted, supplemental oxygen use [at rest/with activity/none], antifibrotic drug use [yes/no]) at enrollment. Associations were considered significant if FDR-corrected *p* ≤ 0.05.

The linearity assumption was evaluated for each protein and outcome combination using natural cubic splines. The proportional hazards assumption was evaluated using weighted Schoenfeld residuals in unadjusted analyses. A threshold of *p* < 0.0001 was used to determine violations. Proteins that failed the linearity assumption were evaluated using two linear piece-wise splines.

To identify predictors that best discriminated the risk of the outcomes and to assess the robustness of the predictive models, we split the IPF cohort into a training set (75%) and test set (25%). Multivariable analyses were performed using Cox regression modeling with the elastic net penalty in the training set. First, both proteins and clinical factors (sex, age, FVC % predicted, DL_CO_ % predicted, oxygen use at rest, oxygen use with activity, antifibrotic drug use) at enrollment were considered as potential predictors. Next we fit a model considering clinical factors only. Model performance in the training and test sets was assessed using Harrell’s C-index and calibration-in-the-large at multiple follow-up times.

## Results

### Cohort characteristics at enrollment

Samples were analyzed from 300 patients with IPF and 100 controls. Two samples (one IPF, one control) were excluded due to quality control issues, thus the analysis cohorts comprised 299 IPF patients and 99 controls. In the IPF cohort, median (Q1, Q3) age was 70.0 (65.0, 75.0) years, 74.6% were men, 95.0% were white, 67.9% were ever smokers (Table 1). Median (Q1, Q3) FVC % predicted was 69.7 (60.9, 80.2) and DL_CO_ % predicted was 40.5 (31.6, 49.4). At the time the blood sample was drawn, 47.5% of patients were taking nintedanib or pirfenidone according to their medical record. In the control cohort, median (Q1, Q3) age was 66.0 (63.0, 71.0) years, 73.7% were men, all were white, 67.7% were ever smokers (Table 1).

**Table 1:** Cohort characteristics at enrollment

	IPF (*n* = 299)	Control (*n* = 99)
Age (years)	70 (65, 75)	66 (63, 71)
Male	223 (74.6%)	73 (73.7%)
Hispanic or Latino ethnicity	8/298 (2.7%)	0
Race		
White	284 (95.0%)	99 (100.0%)
Black or African-American	7 (2.3%)	0
Asian	5 (1.7%)	0
Other	3 (1.0%)	0
Smoking status		
Current	2 (0.7%)	0 (0.0%)
Past	201 (67.2%)	67 (67.7%)
Never	96 (32.1%)	32 (32.3%)
Diagnostic criteria [13]		
Definite IPF	221 (73.9%)	–
Probable IPF	62 (20.7%)	–
Possible IPF	16 (5.4%)	–
FVC % predicted	69.7 (60.9, 80.2)	–
DL_CO_ % predicted	40.5 (31.6, 49.4)	–
Antifibrotic therapy		–
Pirfenidone	97 (32.4%)	–
Nintedanib	45 (15.1%)	–
Statin use	160 (53.5%)	49/99 (49.5%)
Medical history		
Coronary artery disease	91 (30.4%)	21 (21.2%)
Diabetes	58 (19.4%)	27 (27.3%)
COPD/emphysema	54 (18.1%)	-
Atrial fibrillation	37 (12.4%)	13/98 (13.3%)
Supplemental oxygen use		
At rest	61/298 (20.5%)	–
With activity only	48/298 (16.1%)	–

Data are median (Q1, Q3), *n* (%) or n/N with available data (%)

*COPD* Chronic obstructive pulmonary disease, *DL*_*CO*_ Diffusing capacity of the lungs for carbon monoxide, *FVC* Forced vital capacity, *IPF* Idiopathic pulmonary fibrosis

### Differentially abundant proteins in patients with IPF compared to controls

**Fig. 1:**
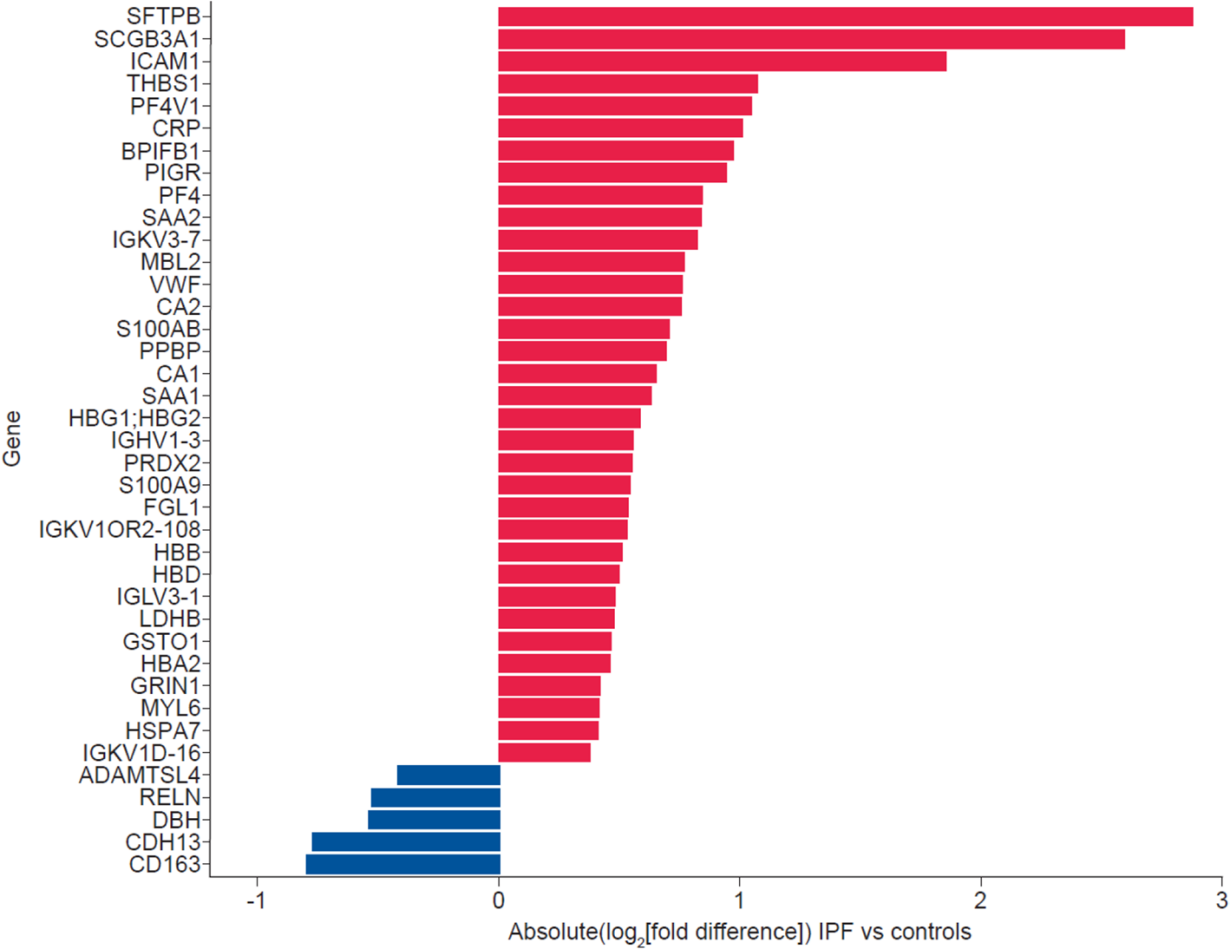
Protein groups with different abundances between the IPF and control cohorts. Protein groups with FDR-corrected *p* ≤ 0.05 and ≥ 1.3-fold difference (absolute[log_2_(fold difference)]≥ $\pm$ 0.38) in abundance in the IPF versus control cohorts at enrollment are shown. Protein groups are labeled by major corresponding gene name

**Fig. 2:**
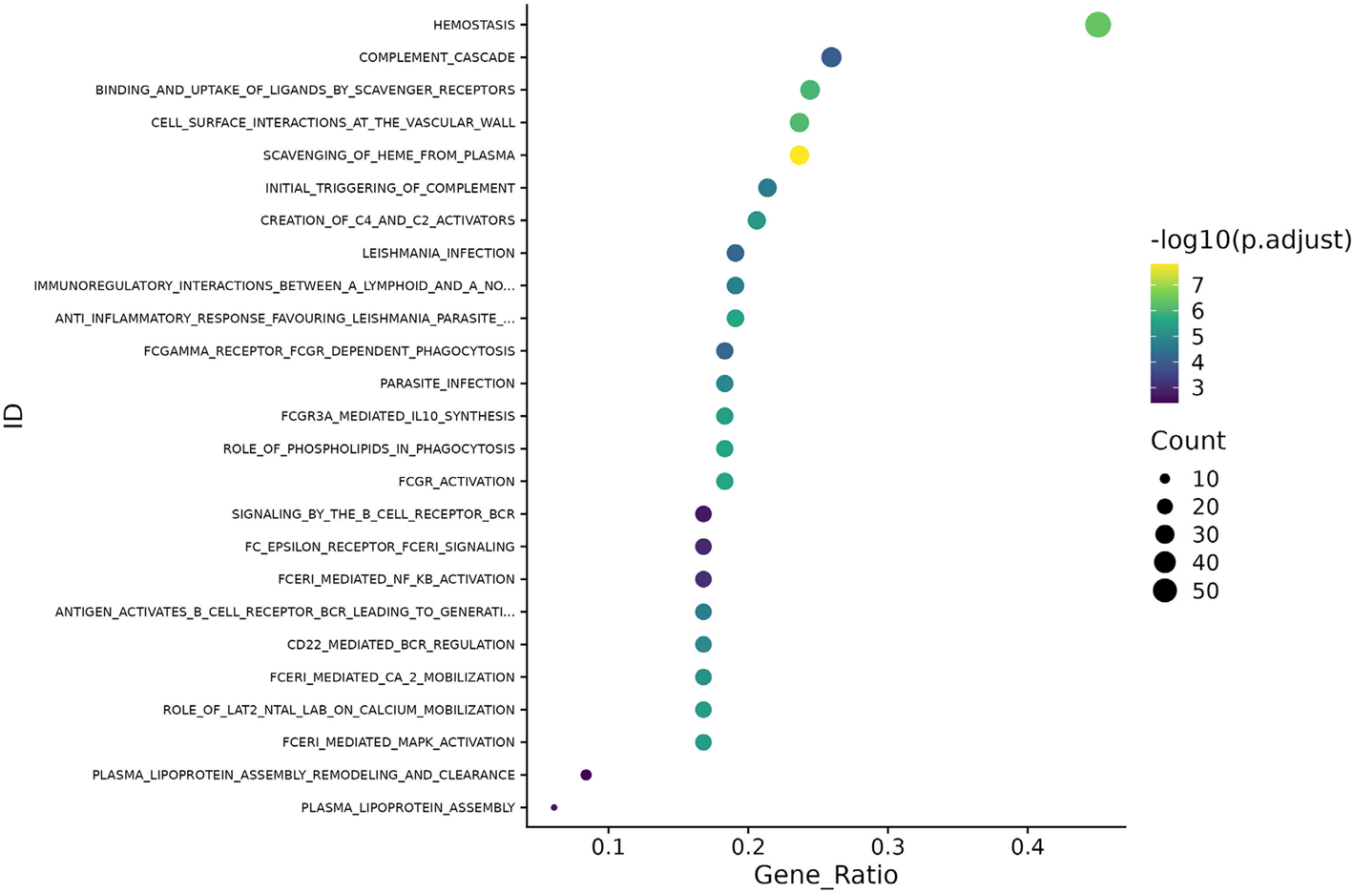
Enriched pathways in the IPF cohort versus control cohort (FDR-corrected *p* < 0.01) ranked by gene ratio. BCR, B-cell receptor. FCERI, Fc epsilon receptor I. FCGR, Fc-gamma receptor

Overall, 761 protein groups corresponding to 736 unique genes were detected. Of these, 168 protein groups were significantly different in abundance in the IPF versus control cohorts, of which 21 were ≥ 1.5-fold different (absolute[log_2_(fold difference)] ≥ + 0.58) and 39 were ≥ 1.3-fold different (absolute[log_2_(fold difference)] ≥ + 0.38) (Fig. 1). Among the top proteins with greater abundance in the IPF versus control cohorts were two lung epithelial proteins, surfactant protein B (SFTPB) and secretoglobin family 3 A member 1 (SCGB3A1), the glycoproteins intercellular adhesion molecule 1(ICAM1) and thrombospondin 1 (THBS1), and the anti-angiogenic chemokine platelet factor 4 variant 1 (PF4v1). Among the top proteins with lower abundance in the IPF cohort than in the controls were CD163, a hemoglobin scavenger receptor; cadherin 13 (CDH13), which regulates axonal growth and protects vascular endothelial cells from apoptosis during oxidative stress; dopamine beta hydroxylase (DBH), which catalyzes the hydroxylation of dopamine to norepinephrine; reelin (RELN), an extracellular matrix serine protease with roles in microtubule function and neuronal migration; and a disintegrin and metalloproteinase with thrombospondin motifs like 4 (ADAMTSL4), which has roles in regulating fibrillin microfibrils and transforming growth factor (TGF)-beta activation. Pathways analyses showed enrichment in pathways associated with hemostasis, the complement cascade, and cell surface interactions at the vascular wall (Fig. 2). Differences in protein abundance between patients with IPF and controls were similar in subsets taking nintedanib, pirfenidone and no antifibrotic therapy (Additional File 2: Fig. S3). Some individual proteins were statistically significantly different in abundance between IPF subsets based on use of antifibrotic therapy; however for most proteins the fold difference was small (Additional File 2: Fig. S4).

### Circulating proteome and measures of disease severity at enrollment

**Fig. 3:**
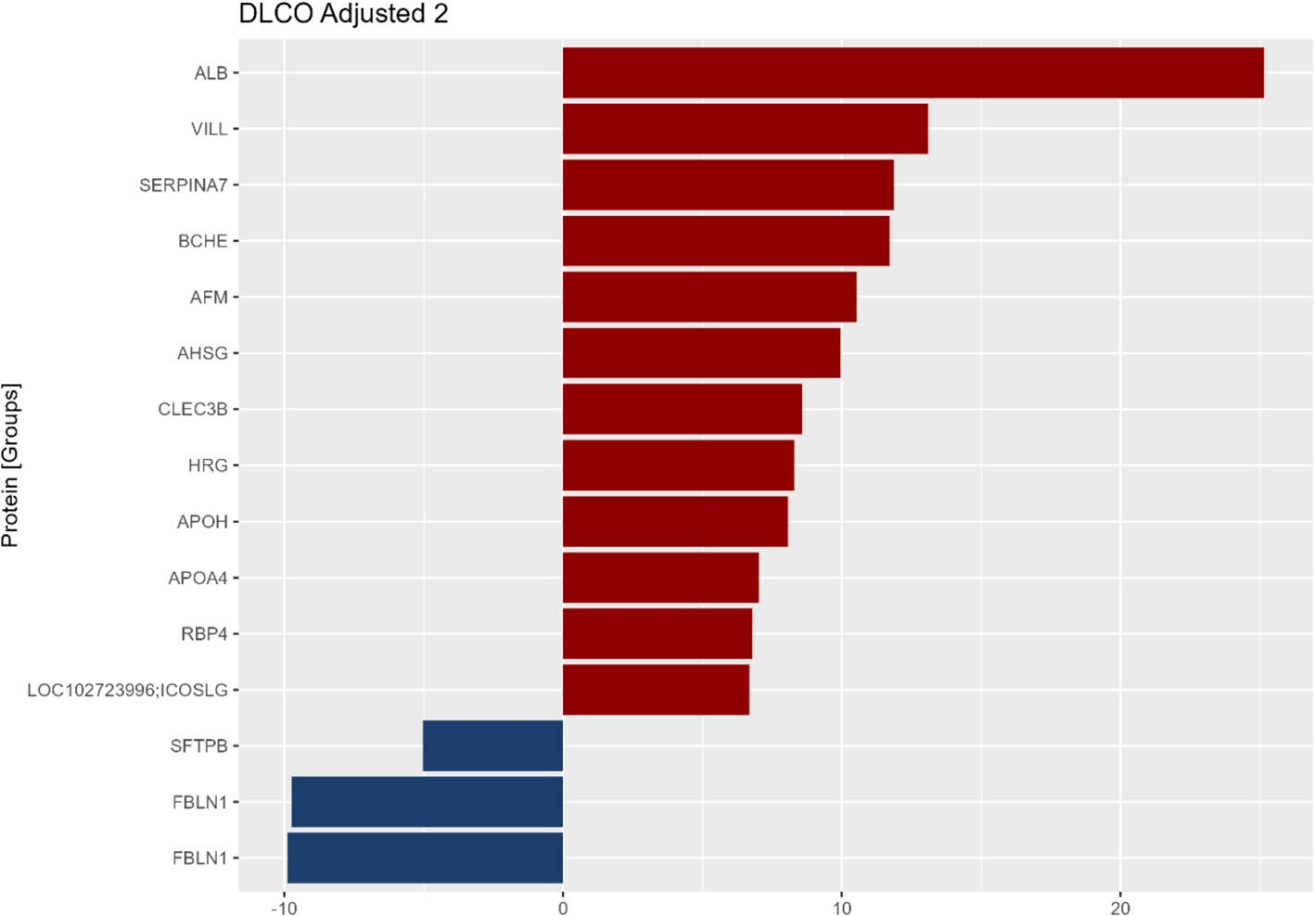
Protein groups statistically significantly associated with DL_CO_ % predicted at enrollment in patients with IPF. Protein groups with FDR-corrected *p* ≤ 0.05 and ≥ 5-unit difference in disease severity measure per twice the protein concentration at enrollment in analyses adjusted for antifibrotic therapy and smoking status are shown. Gene names corresponding to major protein IDs are shown. The two protein groups related to the gene FBLN1 represent unique protein groupings based on the detected peptides (Uniprot ID P23142 and B1AHL2)

Proteins statistically or clinically significantly associated with FVC or DLCO % predicted at enrollment are shown in Tables S1 and S2. While ApoA1 was the only protein that was statistically significantly associated with FVC % predicted at enrollment, many other proteins had large, clinically significant effect sizes (Table S1) Patients with higher levels of ApoA1 had higher FVC % predicted (difference in FVC % predicted per unit higher log_2_ ApoA1: 20.1; *p* = 0.007). Associations were largely unchanged after adjustment for antifibrotic therapy use (Additional File 2: Table S1). In analyses adjusted for antifibrotic therapy use and smoking status, fifteen proteins were significantly associated with DL_CO_ % predicted at enrollment (Fig. 3). Patients with higher levels of the extracellular matrix protein fibulin 1 (FBLN1) and the lung epithelial protein SFTPB tended to have lower DL_CO_ % predicted, while those with higher levels of albumin (ALB), villin like protein (VILL) and SERPINA7 tended to have higher DL_CO_ % predicted (Additional File 2: Table S2).

### Circulating proteome and disease progression

Over a median follow-up of 39.9 months, 221 patients met the composite of decline in FVC % predicted ≥ 10%, death, or lung transplant (127 FVC declines, 81 deaths, 13 lung transplants). In unadjusted univariable analyses, 14 proteins were significantly associated with this outcome (Additional File 2: Fig. S5A). After adjustment for clinical covariates, five proteins remained significantly associated with this outcome (Additional File 2: Fig. S5B). The elastic net did not select any variables with an absolute coefficient > 0.001.

**Fig. 4:**
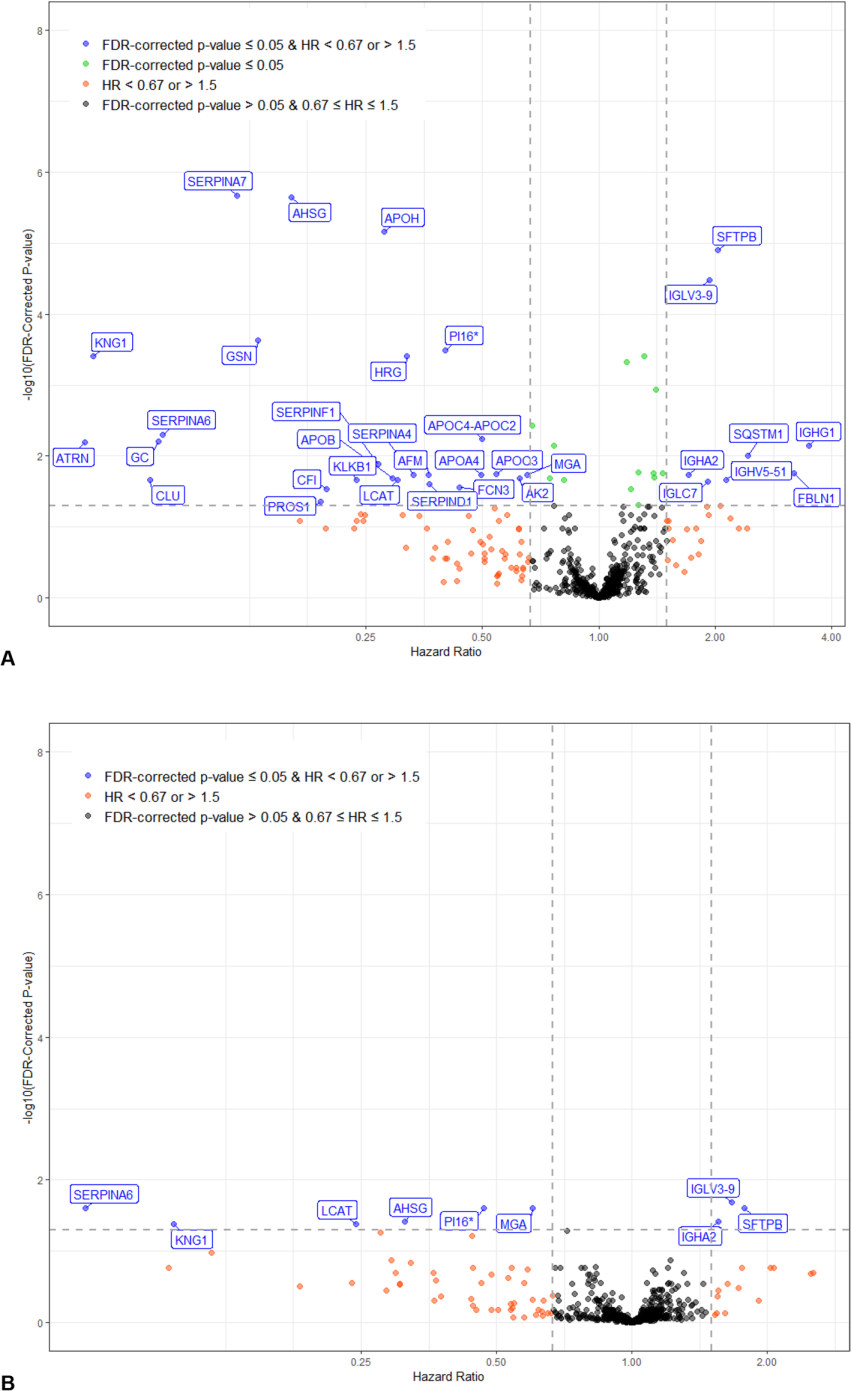
Associations between proteins and respiratory death or lung transplant in patients with IPF. **A** Univariable analyses unadjusted for clinical covariates and **B** univariable analyses adjusted for clinical covariates at enrollment

**Fig. 5:**
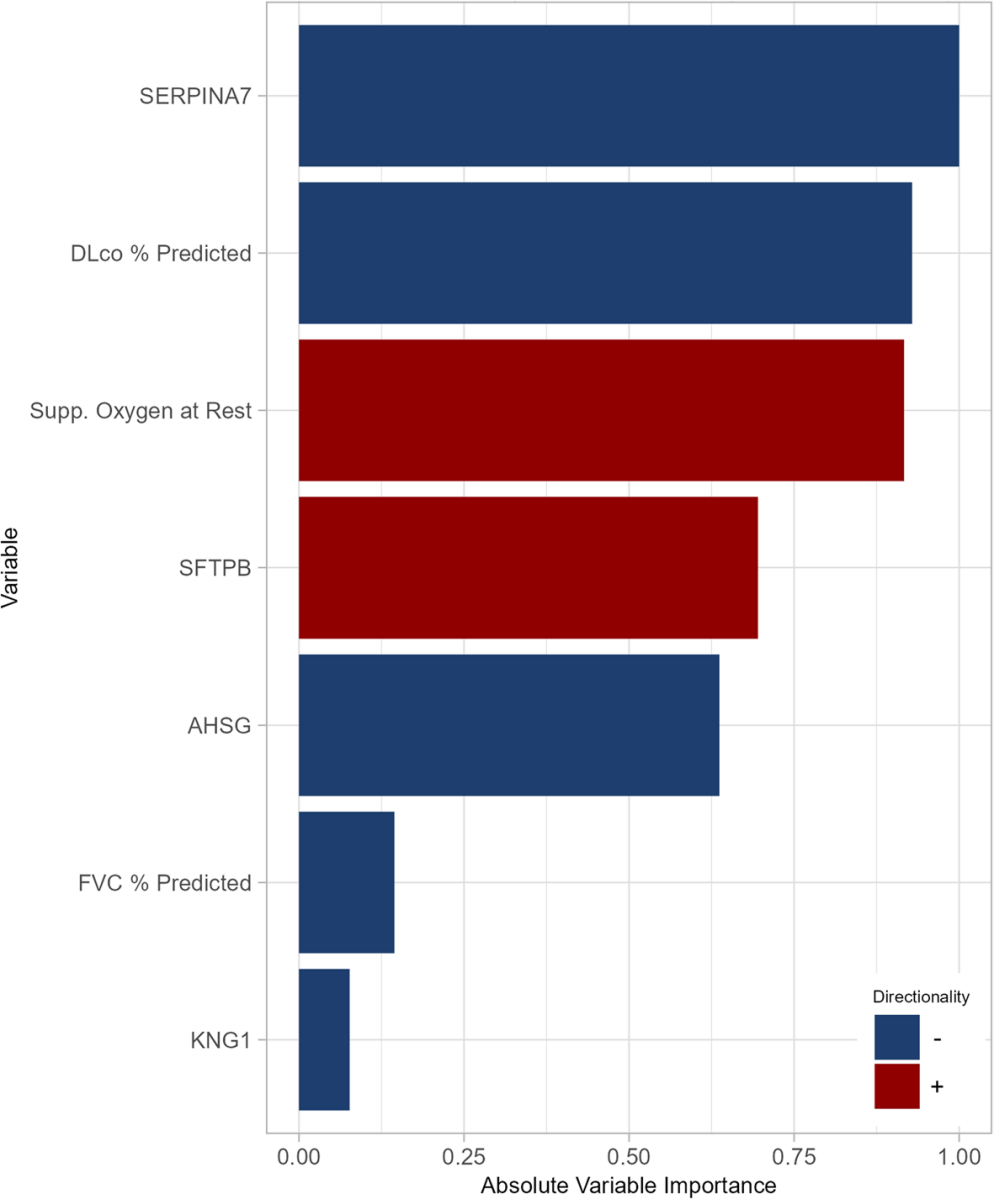
Variable importance of predictors of respiratory death or lung transplant in patients with IPF. Three clinical factors and four proteins selected as predictors of respiratory death or lung transplant using a multivariable model in the training set are shown. SERPINA7, SFTPB, alpha 2 HS glycoprotein and kininogen

The composite of respiratory death or lung transplant was met by 145 patients (104 respiratory deaths, 41 lung transplants). A further 33 patients had non-respiratory deaths. In unadjusted univariable analyses, 47 proteins were significantly associated with the risk of respiratory death or lung transplant (Fig. 4A). After adjustment for clinical covariates, nine proteins remained significantly associated with this outcome (Fig. 4B). In multivariable analyses considering proteins and clinical factors, the elastic net model selected three clinical factors and four proteins, with a C-index of 0.78 in the training set. The model based on clinical factors only selected three factors and had a C-index of 0.72 in the training set. In the test set, C-indices for models based on proteins plus clinical factors or clinical factors only were 0.72 and 0.72, respectively. Model calibration was good: predicted and observed event probabilities were very similar (Additional File 2: Fig. S6). The variable importance of the predictors selected in the model based on proteins and clinical factors in the training set are shown in Fig. 5. The proteins of greatest importance were SERPINA7, SFTPB, alpha 2 HS glycoprotein (AHSG) and kininogen 1 (KNG1).

## Discussion

This is the first large study to use mass spectrometry to quantify the circulating proteome in a multicenter cohort of patients with IPF. Prior studies have employed proximity extension or aptamer-based assays that may be biased, as they detect only specifically targeted proteins [8, 10, 11]. Thus, this MS-based analysis represents a complementary approach, detecting abundant proteins relevant to IPF that are not covered by existing targeted panels. Our analysis confirmed proteins associated with the presence or progression of IPF in previous studies such as SERPINA7 and AHSG. It also revealed new proteins that provided information about prognosis independent of clinical factors such as KNG1.

The top six proteins that we identified as significantly more abundant in patients with IPF than in controls have previously been associated with pulmonary fibrosis: SFTPB [21], SCGB3A1 [21], ICAM1 [22], THBS1 [8], PF4V1 [8], CRP [23]. Of particular interest was the finding that CD163 was less abundant in patients with IPF than in controls. CD163 is part of the "scavenging of heme from plasma" pathway, which was the pathway most significantly enriched in the IPF versus control cohorts. In total, 31 of 131 genes from that pathway showed differential abundance between the IPF and control cohorts. CD163 is involved in the clearance of hemoglobin/haptoglobin complexes expressed on the surface of cells from the monocyte/macrophage lineage [24]. After shedding, the soluble form has anti-inflammatory functions and may be an indicator of macrophage activation [25]. CD163 gene expression has been shown to be downregulated in lung biopsies from patients with IPF compared to controls [26, 27].

We found the reverse cholesterol transporter ApoA1 was statistically significantly associated with FVC % predicted at enrollment and many other apolipoproteins also were associated with FVC with clinically significant effect sizes, whereby higher apolipoprotein levels were associated with higher FVC at enrollment. Additionally, we identified another reverse cholesterol transport pathway member, lecithin–cholesterol acyltransferase (LCAT), as potentially protective in patients with IPF: patients with higher LCAT levels had a decreased risk of respiratory death or lung transplant. Evidence suggests ApoA1 has anti-inflammatory and anti-oxidative functions and may have a protective role in IPF [28–32]. There is a growing body of data showing that lipid metabolism is affected in IPF. For example, a recent single-cell gene expression analysis demonstrated the cholesterol homeostasis pathway is downregulated in lung tissues from patients with advanced IPF [33]. When considered alongside the broader literature, our findings support the conclusion that there is an inverse correlation between lipoprotein levels and both the severity and progression of IPF.

Our MS-based analyses provide a view into protein abundance in IPF and the association of circulating proteins with mortality risk that is complementary to that provided by targeted proteomic assays in IPF cohorts. While comparing results across these platforms is challenging due to differences in assay methodologies, top hits from these multiple discovery platforms could be combined to curate a set of high-yield proteins to evaluate on quantitative assays that can be more readily standardized and optimized for clinical use. Our deep proteome analysis confirmed the findings of a prior aptamer-based proteomic analysis in this same cohort that SERPINA7 and AHSG are independent markers of mortality risk [34]. More recently, a cluster-aided prediction modeling approach in this cohort that included both circulating proteins and miRNAs demonstrated that SERPINA7 is a robust predictor of clinical outcomes in IPF, including death or lung transplantation [35]. Indeed, in our multivariable model, SERPINA7 was a more important predictor of respiratory death or lung transplant than clinical variables. SERPINA7 is a thyroxine-binding globulin that binds tri-iodothyronine and thyroxine. Previous work has suggested that hypothyroidism is a predictor of mortality in patients with IPF [36]. AHSG is a TGF-β antagonist. Increased SMAD2 phosphorylation and elevated TGF-β-mediated suppression of immune cell function, as shown by inhibition of macrophage activation, have been observed in AHSG-deficient mice [37, 38].

In a previous analysis of plasma concentrations of seven proteins in patients with IPF, levels of three proteins were associated with mortality, with the optimal threshold that predicted survival found to be higher in patients who had been exposed to antifibrotic therapy [39]. In our analyses, the circulating proteome was similar in subsets of patients based on use of antifibrotic therapy and associations between proteins and disease severity measures were largely unchanged after adjustment for antifibrotic therapy at enrollment. This finding is consistent with the aptamer-based proteomics analysis conducted in the same cohort [8].

We chose not to immunodeplete our plasma samples prior to MS-based protein detection and quantification. While the presence of albumin and immunoglobulins can mask signals from low-abundance proteins, albumin also binds many small proteins and peptides. As such, depleting albumin can unintentionally remove these bound molecules, potentially losing valuable biological information and distorting protein quantifications. Instead, we employed a spectrometry-based approach using a deep experimental spectral library. A pool of samples was immunodepleted of the seven most abundant proteins and fractionated twice to capture peptide fragmentation spectra of proteins of lower abundance. Since immunodepletion also depletes signaling proteins and damage-associated molecular patterns (DAMPs), the spectral library was extended with fractionated peptides from two pools of undepleted samples. The use of an experimental spectral library boosted both the total coverage and sample-wise depth. Other strengths of our analyses include the use of multicenter cohorts of patients with IPF and controls and an unbiased approach to analyze the circulating proteome. Limitations include that the methodology was unable to detect proteins of very low abundance such as certain cytokines or chemokines. Data on antifibrotic therapy use was extracted from patient’s medical charts and adherence is unknown. Additionally, information on duration of antifibrotic therapy in those on nintedanib or pirfenidone at enrollment was not ascertained.

In conclusion, mass spectrometry-based proteomic analysis of data from the IPF-PRO Registry confirmed proteins previously associated with the presence, severity and progression of IPF and revealed new proteins for investigation as potential biomarkers. Certain proteins provided information about prognosis independent of clinical factors, suggesting that a biomarker-inclusive model could improve prognostication in patients with IPF. Future analyses will examine changes in the circulating proteome as IPF progresses. Such data are expected to provide insights into the molecular pathways that underlie disease progression, including those that remain active despite antifibrotic therapy.

## Supplementary Information

Supplementary Material 1.

## Data Availability

The datasets analyzed during the current study are not publicly available, but are available from the corresponding author on reasonable request.

## Abbreviations

BCR: B-cell receptor
COPD: Chronic obstructive pulmonary disease
DAMPs: Damage-associated molecular patterns
DL_CO_: Diffusing capacity of the lungs for carbon monoxide
FCERI: Fc epsilon receptor I
FCGR: Fc-gamma receptor
FDR: False discovery rate
FVC: Forced vital capacity
IPF: Idiopathic pulmonary fibrosis
IPF-PRO: Idiopathic pulmonary fibrosis prospective outcomes
LC: Liquid chromatography
MS: Mass spectrometry
MURDOCK: Measurement to understand the reclassification of disease of cabarrus/kannapolis
PCA: Principal component analysis
TGF: Transforming growth factor beta
