# Supplementary material for: Mass spectrometry-based peripheral blood proteomics for biomarker discovery in idiopathic pulmonary fibrosis
Source: Respir Res. 2025 Oct 22;26:294. doi: 10.1186/s12931-025-03377-5 (PMC12548219; doi:10.1186/s12931-025-03377-5)

**Additional file 1**

**Section S1: Methods**

***Sample preparation***

Plasma samples were randomized based on patient ID to mitigate plate-wise batch effects. Liquid handling was performed on the Agilent Bravo platform in 96-well format. Of each sample, 1uL was reduced and alkylated with 24 uL 10mM Tris(2-chloroethyl) phosphate, 40 mM Chloroacetamide, 100 mM Tris/HCl (pH 8.5) at 95 °C for 10 min. Samples were cooled on ice for 5 mins before proteins were digested with 7.5 uL Trypsin (0.5 ug/uL, Sigma #T6567) and 7.5 uL LysC (0.5 ug/uL, Wako Chemicals #129-02541) at 37°C for 4 hours. Samples were acidified with 60 uL 0.2% Trifluoroacetic acid before loading 1 uL onto Evotip disposable C18 trap columns (Evosep, #EV2011 Evotip Pure) according to the manufacturer’s recommendations.

To boost peptide identification during downstream data processing, we generated a deep experimental spectral library consisting of three fractionated sample pools: one from plasma samples of both patients with IPF and controls that was depleted of the seven most abundant proteins (albumin, immunoglobulin A, immunoglobulin G, transferrin, haptoglobin, antitrypsin, fibrinogen) before digestion, and two from digested peptides from samples of either patients with IPF or controls. To this end, 1uL plasma from 100 randomly selected samples (from patients with IPF and controls) were pooled and depleted of the top seven most abundant proteins using an affinity spin cartridge (Agilent, #5188-6408). Proteins were reduced, alkylated and digested as described above. Digested peptides were fractionated in technical duplicates into 24 fractions each by high-pH C18 reversed phase nanoflow UHPLC (Easy-nLC 1200, Thermo Scientific) using a 30 cm in-house packed column (Composite Metal Service Ltd., #TSP075375) containing ReproSil-Pur 120 C18-AQ 1.9 µm resin (Dr. Maisch GmbH, #r119.aq). Peptides were separated using a binary buffer system with Buffer A (pH 10 with ~5 mM ammonium formate) and Buffer B (80% Acetonitrile (AcN), pH 10 with ~ 5 mM ammonium formate) in a 120 min non-linear gradient: 2-6% Buffer B for 1 min, 6-32% for 102 min, 32-55% for 9 min, 55-95% for 2 min and 95% for 5 min. The eluate was concatenated into 24 fractions in 30 second intervals. Peptide concentrations were determined by nanodrop and 500 ng of each fraction was loaded onto Evotips. Two additional experimental spectral libraries were created from pools of peptides taken from digested samples, each containing exclusively IPF or exclusively control samples. The peptide pools were fractionated and loaded onto Evotips as described above. The use of an experimental spectral library boosted total coverage and sample-wise depth. The average depth increased from 384.8 in library-free mode to 409.1 using the experimental spectral library, and the number of protein groups identified increased from 434 to 568 (**Figure S1**).

***Liquid chromatography coupled to tandem mass spectrometry (LC-MS/MS)***

Peptides were analyzed by LC coupled to tandem mass spectrometry (MS/MS) for identification and quantification. LC was performed on an Evosep One (Evosep, #EV-1000) using a standardized 44-minute, non-linear gradient (“30 SPD”) [1] and peptides were separated using a 15 cm in-house packed C18-reversed phase column (Composite Metal Service Ltd., #TSP075375) containing ReproSil-Pur 120 C18-AQ 1.9 µm resin (Dr. Maisch GmbH, #r119.aq). Column oven (Sonation GmbH) temperature was set to 60°C and monitored using SprayQC (Scheltema and Mann, 2012). Eluting peptides were analyzed using an Exploris Orbitrap 480 mass spectrometer (Thermo Scientific) coupled to the LC with a nanoelectronspray ion online source. Two samples (one IPF, one control) were excluded due to quality control issues.

All experimental spectral library samples were acquired in data-dependent (DDA), positive ion mode using a survey scan at a resolution of 120000 ranging from 350 to 1400 m/z at a normalized AGC target of 300%. The maximum injection time was set to 45 ms. The top 15 highest intensity precursor ions with 2-5 charges were isolated for higher-energy collisional dissociation (HCD) using 1.2 m/z wide isolation windows and 27% HCD collision energy. The resulting fragment ion spectra were acquired at a resolution of 15000 ranging from 350 to 1000 m/z at a normalized AGC target of 1000%. Maximum injection time was set to 22 ms. Dynamic exclusion of previously fragmented ions was set to 30 s to reduce redundant peak picking.

Cohort samples were acquired in data-independent (DIA), positive ion mode. Survey scan settings were identical to those described for DDA above. Precursor ions were isolated for HCD using 22 evenly spaced, 13.7 m/z wide windows between 361-1033 m/z with a window overlap of 1 m/z. Orbitrap resolution, HCD collision energy, AGC target and maximum injection time were identical to DDA settings described above.

***LC-MS/MS data analysis***

Peptide identification and spectral matching from DDA MS raw files of the experimental spectral library samples were performed with the computational proteomics platform MaxQuant [2] version 1.6.14, using standard settings. Protein inference was performed using the UniProt database reference proteome (Uniprot, UP000005640_9606 and UP000005640_9606_additional). Digestion mode was set to specific for Trypsin/P, the minimum peptide length was set to 7 and a maximum of two missed cleavages were allowed. Cysteine carbamidomethylation was specified as a fixed modification, while methionine oxidation and acetylation of the N-terminus were set as variable modifications. False discovery rates (FDR) on both peptide and protein level were kept at 1%. An experimental spectral library was generated from the MaxQuant output files and the corresponding MS raw files using Spectronaut (Biognosis) version 14.

Protein identification and quantification from DIA MS raw files of the cohort samples were also performed with Spectronaut. Spectral matching and peptide identification were performed in library mode using the deep experimental spectral library. BGS factory settings were used and label-free quantification was performed with QUANT 2.0. The results were log_2_ transformed and missing values were imputed sample-wise by downshift imputation from a normal distribution whose standard deviation was 0.3 times the standard deviation of the log_2_ protein group intensity distribution, and whose mean was 1.8 standard deviations below the mean of protein group intensities. Batch correction was performed with the empirical Bayes method in Python using pyComBat after grouping samples by plate number. Principal component analysis (PCA) after batch correction showed good clustering of the reference samples (**Figure S2**).

**Figure S1.** Increase in proteome coverage by using an experimental spectral library, tested on a subset of the full cohort (Plate 1). Histograms of sample depths of cohort files processed in (**A**) Spectronaut direct (= library-free) mode or in (**B**) Spectronaut library mode with a deep experimental spectral library.

**
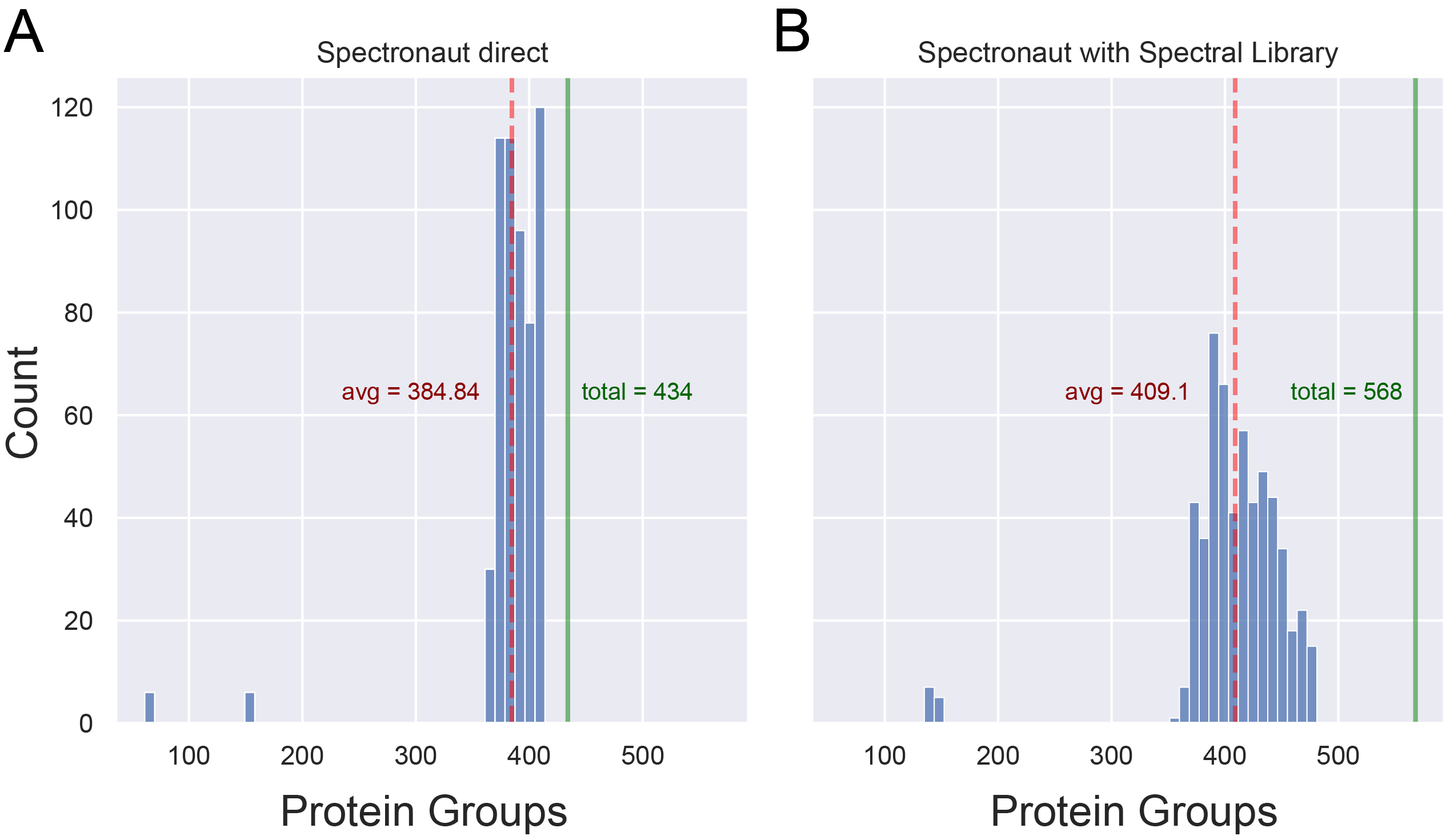
**

**Figure S2.** Principal component analysis (PCA) plot of samples after batch correction.


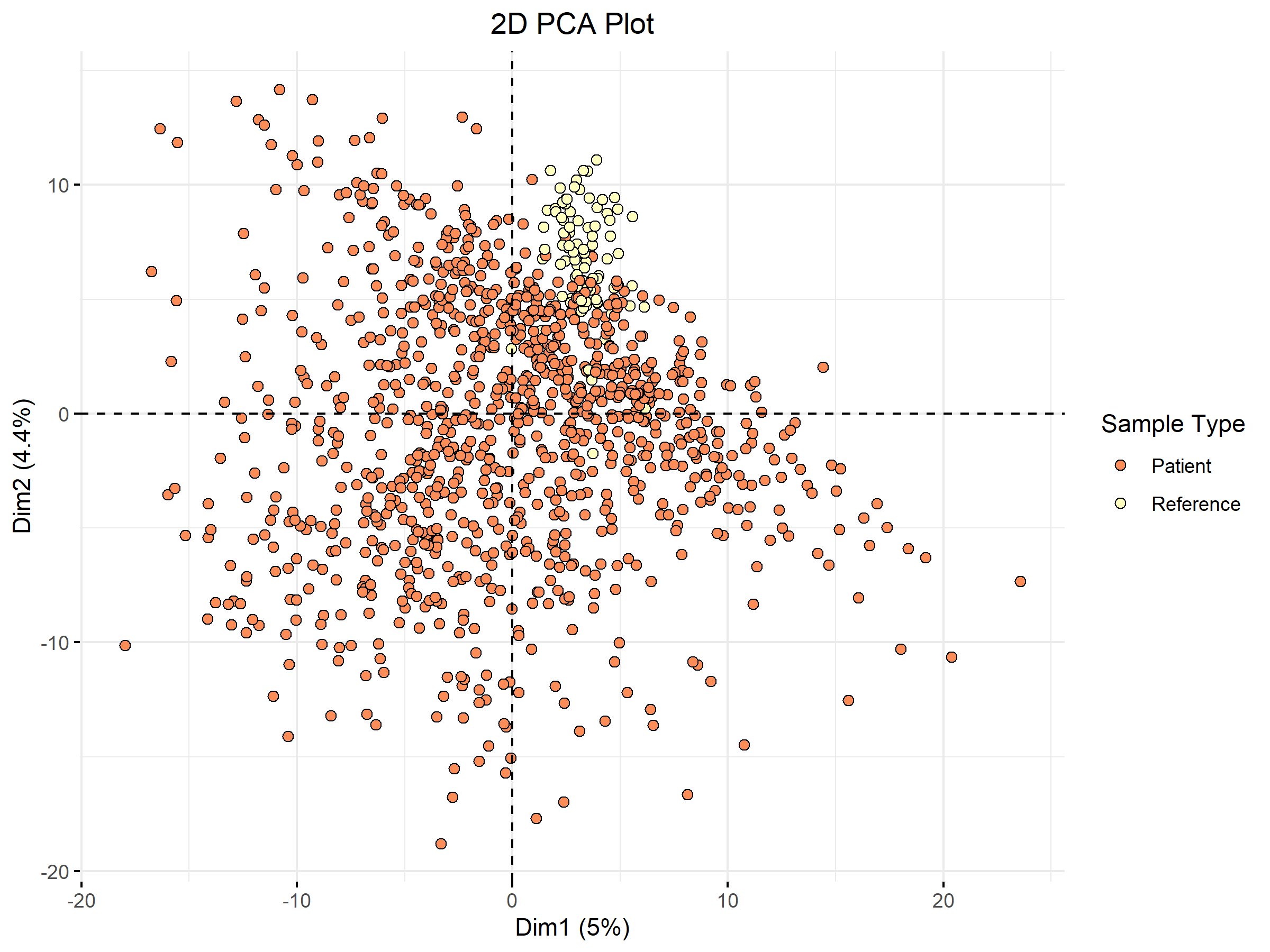


**References**

1. Bache N, Geyer PE, Bekker-Jensen DB, et al. A novel LC system embeds analytes in pre-formed gradients for rapid, ultra-robust proteomics. Mol Cell Proteomics 2018;17:2284–2296.
2. Cox J, Mann M. MaxQuant enables high peptide identification rates, individualized p.p.b.-range mass accuracies and proteome-wide protein quantification. Nat Biotechnol 2008;26:1367–1372.

**Additional File 2**

**Table S1.** Proteins statistically or clinically significantly associated with FVC % predicted at enrollment (FDR-corrected *p-v*alue ≤0.05 or > 5-unit difference in the disease severity measure per unit difference in log_2_ protein abundance *i.e.,* twice the protein concentration) at enrollment in unadjusted or adjusted analyses.

|  | | **Unadjusted** | | | **Adjusted for Antifibrotic Treatment Use at Enrollment** | | |
| --- | --- | --- | --- | --- | --- | --- | --- |
| **Uniprot** | **Gene** | **Difference*** | **P-value** | **FDR- Corrected P-value** | **Difference*** | **P-value** | **FDR- Corrected P-value** |
| P02768 | ALB | 24.6019 | 0.001 | 0.095 | 24.7415 | 0.001 | 0.097 |
| P01008 | SERPINC1 | 22.2012 | 0.001 | 0.077 | 23.0346 | 0.001 | 0.068 |
| P02647 | APOA1 | 20.0646 | 0.000 | 0.007 | 20.5655 | 0.000 | 0.005 |
| P08697 | SERPINF2 | 18.3307 | 0.011 | 0.160 | 18.8274 | 0.009 | 0.161 |
| P10909-6 | CLU | 17.2143 | 0.037 | 0.287 | 17.9611 | 0.031 | 0.264 |
| P19827 | ITIH1 | 17.1312 | 0.006 | 0.147 | 17.8169 | 0.004 | 0.128 |
| P01042 | KNG1 | 15.1038 | 0.035 | 0.274 | 15.0417 | 0.036 | 0.282 |
| D6RF35;P02774;P02774-3 | GC | 14.9189 | 0.046 | 0.312 | 14.9863 | 0.046 | 0.313 |
| A0A182DWH7;P49908 | SELENOP | 12.9808 | 0.013 | 0.169 | 13.0305 | 0.014 | 0.175 |
| P08185 | SERPINA6 | 12.4878 | 0.111 | 0.434 | 12.4171 | 0.114 | 0.441 |
| P02652;V9GYM3 | APOA2 | 12.2734 | 0.002 | 0.099 | 12.4296 | 0.002 | 0.097 |
| P06396 | GSN | 11.5485 | 0.017 | 0.188 | 11.4388 | 0.019 | 0.203 |
| A0A087WSY5;Q96IY4 | CPB2 | 11.4626 | 0.012 | 0.169 | 11.6169 | 0.012 | 0.170 |
| P02787 | TF | 11.3194 | 0.074 | 0.375 | 11.1496 | 0.080 | 0.395 |
| O95445 | APOM | 11.1782 | 0.005 | 0.147 | 11.5704 | 0.004 | 0.128 |
| P80108 | GPLD1 | 10.7526 | 0.001 | 0.077 | 11.8440 | 0.001 | 0.058 |
| O75882;O75882-2;O75882-3 | ATRN | 10.5536 | 0.236 | 0.596 | 10.5291 | 0.239 | 0.599 |
| P04180 | LCAT | 10.3923 | 0.007 | 0.159 | 10.4970 | 0.007 | 0.161 |
| Q14520;Q14520-2 | HABP2 | 10.2146 | 0.017 | 0.188 | 10.5016 | 0.015 | 0.181 |
| P19823 | ITIH2 | 10.0359 | 0.070 | 0.364 | 10.3294 | 0.064 | 0.361 |
| P29622 | SERPINA4 | 9.7460 | 0.013 | 0.169 | 9.7282 | 0.014 | 0.175 |
| A0A0S2Z4L3;A0A3B3ISJ1;P07225 | PROS1 | 9.7174 | 0.116 | 0.439 | 9.9007 | 0.111 | 0.439 |
| P05160 | F13B | 8.9700 | 0.051 | 0.324 | 8.9678 | 0.052 | 0.323 |
| P25311 | AZGP1 | 8.9137 | 0.005 | 0.147 | 8.9005 | 0.006 | 0.150 |
| P02753;Q5VY30 | RBP4 | 8.7653 | 0.001 | 0.071 | 8.9075 | 0.001 | 0.063 |
| P04196 | HRG | 8.5487 | 0.004 | 0.132 | 8.7574 | 0.003 | 0.128 |
| P00488 | F13A1 | 8.4580 | 0.010 | 0.159 | 8.3939 | 0.011 | 0.166 |
| P00747 | PLG | 8.4507 | 0.189 | 0.542 | 8.8985 | 0.170 | 0.526 |
| K7ERI9;P02654 | APOC1 | 8.3515 | 0.005 | 0.139 | 8.5920 | 0.004 | 0.128 |
| P06276 | BCHE | 8.1465 | 0.028 | 0.248 | 8.3361 | 0.028 | 0.253 |
| P20851;P20851-2 | C4BPB | 8.0394 | 0.054 | 0.328 | 8.0663 | 0.054 | 0.326 |
| P00740 | F9 | 7.9278 | 0.066 | 0.358 | 8.2298 | 0.059 | 0.346 |
| P06727 | APOA4 | 7.6856 | 0.001 | 0.071 | 7.9813 | 0.000 | 0.058 |
| P02760 | AMBP | 7.6788 | 0.056 | 0.329 | 7.5703 | 0.061 | 0.354 |
| H0YAC1;P03952 | KLKB1 | 7.5399 | 0.139 | 0.474 | 7.6613 | 0.135 | 0.471 |
| P05543 | SERPINA7 | 7.5279 | 0.065 | 0.358 | 7.7153 | 0.060 | 0.352 |
| P04217-2 | A1BG | 7.1985 | 0.061 | 0.349 | 7.9569 | 0.044 | 0.312 |
| P04004 | VTN | 7.0988 | 0.048 | 0.313 | 7.3555 | 0.042 | 0.312 |
| P02749 | APOH | 7.0051 | 0.014 | 0.173 | 7.1108 | 0.013 | 0.173 |
| P27169 | PON1 | 6.9416 | 0.008 | 0.159 | 7.1007 | 0.007 | 0.161 |
| A0A0C4DFP6;Q9NQ79;Q9NQ79-2;Q9NQ79-3 | CRTAC1 | 6.6659 | 0.002 | 0.107 | 6.7932 | 0.002 | 0.097 |
| P05546 | SERPIND1 | 6.6284 | 0.095 | 0.415 | 7.0426 | 0.080 | 0.395 |
| P04217 | A1BG | 6.6282 | 0.212 | 0.566 | 7.4326 | 0.173 | 0.526 |
| P05155;P05155-3 | SERPING1 | 6.4510 | 0.065 | 0.358 | 6.4780 | 0.064 | 0.361 |
| A0A087WT59;P02766 | TTR | 6.4155 | 0.082 | 0.389 | 6.5412 | 0.079 | 0.395 |
| P55058 | PLTP | 6.3131 | 0.095 | 0.415 | 6.3634 | 0.095 | 0.419 |
| B0YIW2;P02656 | APOC3 | 6.2599 | 0.001 | 0.085 | 6.4780 | 0.001 | 0.073 |
| P02765 | AHSG | 6.0975 | 0.082 | 0.389 | 6.1061 | 0.082 | 0.395 |
| P15169 | CPN1 | 6.0862 | 0.247 | 0.610 | 6.4272 | 0.225 | 0.583 |
| P02745 | C1QA | 6.0642 | 0.170 | 0.511 | 5.9884 | 0.177 | 0.526 |
| P00734 | F2 | 6.0588 | 0.284 | 0.650 | 6.1109 | 0.285 | 0.649 |
| P00450 | CP | 6.0277 | 0.171 | 0.511 | 5.9356 | 0.180 | 0.526 |
| P36955 | SERPINF1 | 5.7555 | 0.140 | 0.476 | 5.7765 | 0.141 | 0.485 |
| G3V2W1;Q9UK55 | SERPINA10 | 5.7551 | 0.053 | 0.327 | 5.9205 | 0.050 | 0.320 |
| P02790 | HPX | 5.5929 | 0.176 | 0.518 | 5.4889 | 0.188 | 0.540 |
| P00748 | F12 | 5.4967 | 0.025 | 0.233 | 5.6966 | 0.021 | 0.214 |
| P04003 | C4BPA | 5.4375 | 0.166 | 0.511 | 5.5772 | 0.159 | 0.503 |
| P08571 | CD14 | 5.3799 | 0.171 | 0.511 | 5.3864 | 0.176 | 0.526 |
| C9JF17;P05090 | APOD | 5.3709 | 0.024 | 0.233 | 5.3479 | 0.025 | 0.242 |
| Q13790 | APOF | 5.2670 | 0.052 | 0.324 | 5.3670 | 0.050 | 0.320 |
| P06681 | C2 | 5.2505 | 0.208 | 0.566 | 5.3440 | 0.202 | 0.560 |
| O15195;O15195-2 | VILL | 5.1526 | 0.179 | 0.522 | 5.0493 | 0.195 | 0.551 |
| P04114 | APOB | 5.1302 | 0.199 | 0.561 | 5.4100 | 0.182 | 0.527 |
| P01019 | AGT | 5.1268 | 0.101 | 0.427 | 5.1099 | 0.103 | 0.433 |
| K7ER74 | APOC4-APOC2 | 4.9482 | 0.014 | 0.177 | 5.0629 | 0.013 | 0.175 |
| F8VZY9;P05783 | KRT18 | -5.0857 | 0.004 | 0.132 | -5.1498 | 0.004 | 0.128 |
| A0A075B6K5 | IGLV3-9 | -5.3185 | 0.000 | 0.067 | -5.3067 | 0.000 | 0.058 |
| A0A0G2JRQ6 |  | -5.3751 | 0.008 | 0.159 | -5.3647 | 0.009 | 0.161 |
| A0A5H1ZRQ7;A0M8Q6 | IGLC7 | -5.5766 | 0.008 | 0.159 | -5.7245 | 0.007 | 0.161 |
| P23142 | FBLN1 | -5.9738 | 0.074 | 0.375 | -5.8861 | 0.082 | 0.395 |
| A0A2Q2TTZ9 | IGKV1D-33 | -6.2065 | 0.012 | 0.169 | -6.1757 | 0.013 | 0.173 |
| B1AHL2 | FBLN1 | -6.2297 | 0.091 | 0.403 | -6.3456 | 0.086 | 0.400 |
| P01624 | IGKV3-15 | -6.4155 | 0.007 | 0.159 | -6.3935 | 0.008 | 0.161 |
| P06312 | IGKV4-1 | -7.0295 | 0.016 | 0.188 | -6.9676 | 0.018 | 0.200 |
| P0DOY2 | IGLC2 | -7.1438 | 0.017 | 0.188 | -7.2298 | 0.016 | 0.185 |
| P01023 | A2M | -7.6422 | 0.013 | 0.169 | -8.0579 | 0.009 | 0.161 |
| P01834 | IGKC | -9.2155 | 0.015 | 0.178 | -9.2723 | 0.015 | 0.181 |
| P01857 | IGHG1 | -9.7755 | 0.010 | 0.159 | -9.7781 | 0.010 | 0.166 |

*Difference in disease severity per unit higher log_2_ concentration of protein.

**Table S2.** Proteins statistically or clinically significantly associated with DLCO % predicted at enrollment (FDR-corrected *p-v*alue ≤0.05 or > 5-unit difference in the disease severity measure per unit difference in log_2_ protein abundance *i.e.,* twice the protein concentration) at enrollment in unadjusted or adjusted analyses.

|  | | **Unadjusted** | | | **Adjusted for Antifibrotic Treatment Use at Enrollment** | | | **Adjusted for Antifibrotic Treatment Use and Smoking Status at Enrollment** | | |
| --- | --- | --- | --- | --- | --- | --- | --- | --- | --- | --- |
| **Uniprot** | **Gene** | **Difference*** | **P-value** | **FDR- Corrected P-value** | **Difference*** | **P-value** | **FDR- Corrected P-value** | **Difference*** | **P-value** | **FDR- Corrected P-value** |
| P02768 | ALB | 23.8059 | 0.000 | 0.019 | 23.8280 | 0.000 | 0.019 | 25.1498 | 0.000 | 0.008 |
| P01042 | KNG1 | 14.8273 | 0.013 | 0.180 | 14.7114 | 0.013 | 0.183 | 15.2161 | 0.009 | 0.150 |
| P02787 | TF | 14.1550 | 0.007 | 0.136 | 13.7320 | 0.009 | 0.155 | 15.7460 | 0.003 | 0.063 |
| O15195;O15195-2 | VILL | 12.7642 | 0.000 | 0.007 | 12.7440 | 0.000 | 0.008 | 13.0930 | 0.000 | 0.005 |
| P05543 | SERPINA7 | 12.2890 | 0.000 | 0.019 | 12.5533 | 0.000 | 0.019 | 11.8694 | 0.000 | 0.023 |
| A0A0S2Z4L3;A0A3B3ISJ1;P07225 | PROS1 | 11.9445 | 0.020 | 0.243 | 12.4929 | 0.015 | 0.198 | 11.5139 | 0.024 | 0.262 |
| P06276 | BCHE | 11.9349 | 0.000 | 0.011 | 12.3782 | 0.000 | 0.008 | 11.7184 | 0.000 | 0.014 |
| P08697 | SERPINF2 | 11.7766 | 0.049 | 0.400 | 12.8462 | 0.033 | 0.320 | 13.8385 | 0.020 | 0.248 |
| A0A182DWH7;P49908 | SELENOP | 11.5319 | 0.008 | 0.143 | 11.6773 | 0.008 | 0.140 | 12.3207 | 0.004 | 0.091 |
| P06396 | GSN | 11.2095 | 0.006 | 0.118 | 10.9256 | 0.007 | 0.131 | 10.7570 | 0.007 | 0.121 |
| P43652 | AFM | 10.5496 | 0.000 | 0.021 | 10.8335 | 0.000 | 0.019 | 10.5328 | 0.000 | 0.021 |
| P02765 | AHSG | 9.9973 | 0.001 | 0.028 | 9.9183 | 0.001 | 0.028 | 9.9460 | 0.000 | 0.024 |
| P19823 | ITIH2 | 9.9557 | 0.031 | 0.307 | 10.5954 | 0.022 | 0.246 | 10.1692 | 0.026 | 0.278 |
| P29622 | SERPINA4 | 9.2689 | 0.005 | 0.105 | 9.2502 | 0.005 | 0.097 | 9.3952 | 0.004 | 0.079 |
| E9PHK0;P05452 | CLEC3B | 8.9424 | 0.001 | 0.034 | 8.9320 | 0.001 | 0.032 | 8.5714 | 0.001 | 0.035 |
| P19827 | ITIH1 | 8.8240 | 0.089 | 0.473 | 9.9401 | 0.057 | 0.432 | 10.1995 | 0.047 | 0.381 |
| P08185 | SERPINA6 | 8.5188 | 0.193 | 0.648 | 8.3518 | 0.202 | 0.648 | 8.9519 | 0.165 | 0.589 |
| P01024 | C3 | 8.4599 | 0.076 | 0.464 | 8.8236 | 0.064 | 0.438 | 8.7583 | 0.062 | 0.418 |
| P08603 | CFH | 8.4284 | 0.040 | 0.359 | 8.5255 | 0.038 | 0.346 | 8.1050 | 0.045 | 0.376 |
| P04196 | HRG | 8.3050 | 0.001 | 0.031 | 8.6725 | 0.000 | 0.020 | 8.2933 | 0.001 | 0.025 |
| P02749 | APOH | 8.2810 | 0.000 | 0.024 | 8.5598 | 0.000 | 0.020 | 8.0700 | 0.001 | 0.024 |
| P01008 | SERPINC1 | 8.1877 | 0.146 | 0.574 | 8.5859 | 0.136 | 0.558 | 10.7913 | 0.058 | 0.412 |
| P10909-6 | CLU | 8.1422 | 0.237 | 0.695 | 9.6629 | 0.163 | 0.581 | 10.6698 | 0.118 | 0.521 |
| P04004 | VTN | 8.0467 | 0.007 | 0.133 | 8.5771 | 0.004 | 0.090 | 8.6302 | 0.003 | 0.078 |
| P05160 | F13B | 7.7725 | 0.043 | 0.365 | 7.8430 | 0.041 | 0.369 | 7.8665 | 0.037 | 0.332 |
| P80108 | GPLD1 | 6.9358 | 0.010 | 0.158 | 7.7061 | 0.007 | 0.131 | 7.8244 | 0.005 | 0.104 |
| O75882;O75882-2;O75882-3 | ATRN | 6.6704 | 0.370 | 0.792 | 6.4245 | 0.387 | 0.789 | 6.5359 | 0.372 | 0.790 |
| Q14520;Q14520-2 | HABP2 | 6.5289 | 0.068 | 0.453 | 7.0710 | 0.049 | 0.401 | 7.2042 | 0.042 | 0.356 |
| P07360 | C8G | 6.4909 | 0.181 | 0.640 | 6.2865 | 0.195 | 0.634 | 6.0904 | 0.202 | 0.620 |
| P00734 | F2 | 6.3417 | 0.179 | 0.637 | 6.6714 | 0.160 | 0.578 | 5.4544 | 0.245 | 0.679 |
| Q6EMK4 | VASN | 6.3115 | 0.085 | 0.468 | 5.7088 | 0.122 | 0.549 | 5.5120 | 0.130 | 0.538 |
| P02753;Q5VY30 | RBP4 | 6.3103 | 0.003 | 0.085 | 6.4035 | 0.003 | 0.085 | 6.7796 | 0.002 | 0.045 |
| A0A087X1L8;K4DIA0;O75144;O75144-2;O75144-3 | ICOSLG | 6.3051 | 0.003 | 0.083 | 6.5051 | 0.002 | 0.065 | 6.6880 | 0.001 | 0.044 |
| P36955 | SERPINF1 | 6.1724 | 0.058 | 0.431 | 6.0037 | 0.066 | 0.438 | 6.7407 | 0.036 | 0.332 |
| P00488 | F13A1 | 6.1164 | 0.026 | 0.277 | 5.9483 | 0.031 | 0.304 | 6.6353 | 0.015 | 0.201 |
| P02647 | APOA1 | 6.1049 | 0.110 | 0.516 | 6.8708 | 0.074 | 0.455 | 6.2155 | 0.101 | 0.489 |
| P00747 | PLG | 5.9536 | 0.268 | 0.728 | 6.9788 | 0.196 | 0.634 | 7.7341 | 0.146 | 0.552 |
| A0A087WT59;P02766 | TTR | 5.7844 | 0.061 | 0.439 | 6.1599 | 0.047 | 0.387 | 6.7831 | 0.026 | 0.283 |
| P07358 | C8B | 5.7705 | 0.161 | 0.600 | 5.2411 | 0.205 | 0.649 | 5.2489 | 0.197 | 0.619 |
| P06727 | APOA4 | 5.7446 | 0.002 | 0.065 | 6.2890 | 0.001 | 0.032 | 7.0269 | 0.000 | 0.014 |
| P13473;P13473-2;P13473-3 | LAMP2 | 5.4117 | 0.098 | 0.504 | 5.6035 | 0.087 | 0.489 | 6.0293 | 0.061 | 0.418 |
| D6RF35;P02774;P02774-3 | GC | 5.3952 | 0.389 | 0.805 | 5.6069 | 0.371 | 0.789 | 5.2533 | 0.394 | 0.797 |
| P02652;V9GYM3 | APOA2 | 5.2742 | 0.108 | 0.516 | 5.4022 | 0.100 | 0.504 | 5.8132 | 0.072 | 0.444 |
| P05546 | SERPIND1 | 5.2323 | 0.115 | 0.527 | 6.0962 | 0.068 | 0.449 | 6.5926 | 0.045 | 0.376 |
| Q96PD5 | PGLYRP2 | 5.1557 | 0.159 | 0.598 | 5.5686 | 0.128 | 0.549 | 5.2865 | 0.143 | 0.552 |
| Q9Y5Y7 | LYVE1 | 5.0752 | 0.004 | 0.096 | 5.2262 | 0.003 | 0.082 | 4.8514 | 0.005 | 0.104 |
| P06681 | C2 | 4.9999 | 0.151 | 0.578 | 5.1200 | 0.141 | 0.558 | 4.7182 | 0.169 | 0.596 |
| Q04756 | HGFAC | 4.7696 | 0.071 | 0.453 | 4.5856 | 0.083 | 0.477 | 5.2730 | 0.043 | 0.365 |
| Q96KN2 | CNDP1 | 4.6448 | 0.003 | 0.074 | 4.6977 | 0.002 | 0.065 | 4.7967 | 0.002 | 0.045 |
| A0A5K1VW67;F8VY04 | AK2 | 3.9676 | 0.005 | 0.115 | 4.1463 | 0.004 | 0.087 | 4.8160 | 0.001 | 0.025 |
| P23381;P23381-2 | WARS1 | 2.8613 | 0.001 | 0.042 | 2.9300 | 0.001 | 0.032 | 2.9174 | 0.001 | 0.028 |
| F5H8B0;P08709;P08709-2 | F7 | 2.4697 | 0.001 | 0.044 | 2.5365 | 0.001 | 0.034 | 2.6742 | 0.000 | 0.023 |
| P0DJI9 | SAA2 | -1.5538 | 0.000 | 0.002 | -1.5429 | 0.000 | 0.002 | -1.5500 | 0.000 | 0.001 |
| P02741 | CRP | -1.6522 | 0.000 | 0.019 | -1.6261 | 0.000 | 0.020 | -1.5689 | 0.000 | 0.023 |
| Q8TDL5 | BPIFB1 | -1.8456 | 0.001 | 0.042 | -1.7646 | 0.002 | 0.065 | -1.8742 | 0.001 | 0.032 |
| P05109 | S100A8 | -2.2975 | 0.001 | 0.039 | -2.3028 | 0.001 | 0.034 | -2.1712 | 0.002 | 0.045 |
| O75368 | SH3BGRL | -2.6147 | 0.001 | 0.039 | -2.6700 | 0.001 | 0.032 | -2.6816 | 0.001 | 0.025 |
| P0DJI8 | SAA1 | -3.4776 | 0.000 | 0.000 | -3.5125 | 0.000 | 0.000 | -3.5483 | 0.000 | 0.000 |
| Q96QR1 | SCGB3A1 | -3.4980 | 0.000 | 0.000 | -3.4829 | 0.000 | 0.000 | -3.5459 | 0.000 | 0.000 |
| A0A075B6K5 | IGLV3-9 | -4.4075 | 0.000 | 0.019 | -4.3816 | 0.000 | 0.020 | -4.4709 | 0.000 | 0.014 |
| P06702 | S100A9 | -4.4684 | 0.000 | 0.000 | -4.4744 | 0.000 | 0.000 | -4.4086 | 0.000 | 0.000 |
| P07988 | SFTPB | -5.2222 | 0.000 | 0.004 | -5.1335 | 0.000 | 0.006 | -5.0265 | 0.000 | 0.006 |
| P01857 | IGHG1 | -5.2906 | 0.096 | 0.498 | -5.3290 | 0.094 | 0.501 | -5.9678 | 0.057 | 0.406 |
| P06331 | IGHV4-34 | -5.3142 | 0.008 | 0.143 | -5.0497 | 0.012 | 0.180 | -4.9050 | 0.014 | 0.192 |
| P02763 | ORM1 | -5.8656 | 0.004 | 0.096 | -5.7460 | 0.005 | 0.099 | -5.5034 | 0.006 | 0.116 |
| P10643 | C7 | -7.8830 | 0.008 | 0.143 | -8.6124 | 0.004 | 0.090 | -8.5206 | 0.004 | 0.085 |
| P02748 | C9 | -8.2917 | 0.006 | 0.124 | -8.1562 | 0.007 | 0.131 | -7.7997 | 0.009 | 0.148 |
| P19652 | ORM2 | -8.5243 | 0.007 | 0.131 | -8.2848 | 0.009 | 0.148 | -8.0582 | 0.009 | 0.150 |
| P01011 | SERPINA3 | -9.5827 | 0.001 | 0.044 | -9.6620 | 0.001 | 0.040 | -9.1624 | 0.002 | 0.051 |
| P23142 | FBLN1 | -10.098 | 0.000 | 0.019 | -9.9379 | 0.000 | 0.020 | -9.7338 | 0.000 | 0.023 |
| B1AHL2 | FBLN1 | -10.470 | 0.001 | 0.028 | -10.771 | 0.000 | 0.020 | -9.8883 | 0.001 | 0.035 |

*Difference in disease severity per unit higher log_2_ concentration of protein.

**Figure S3**. Differences in protein abundance between controls and patients with IPF in subsets by antifibrotic treatment status at enrollment compared with the overall cohort of patients with IPF.


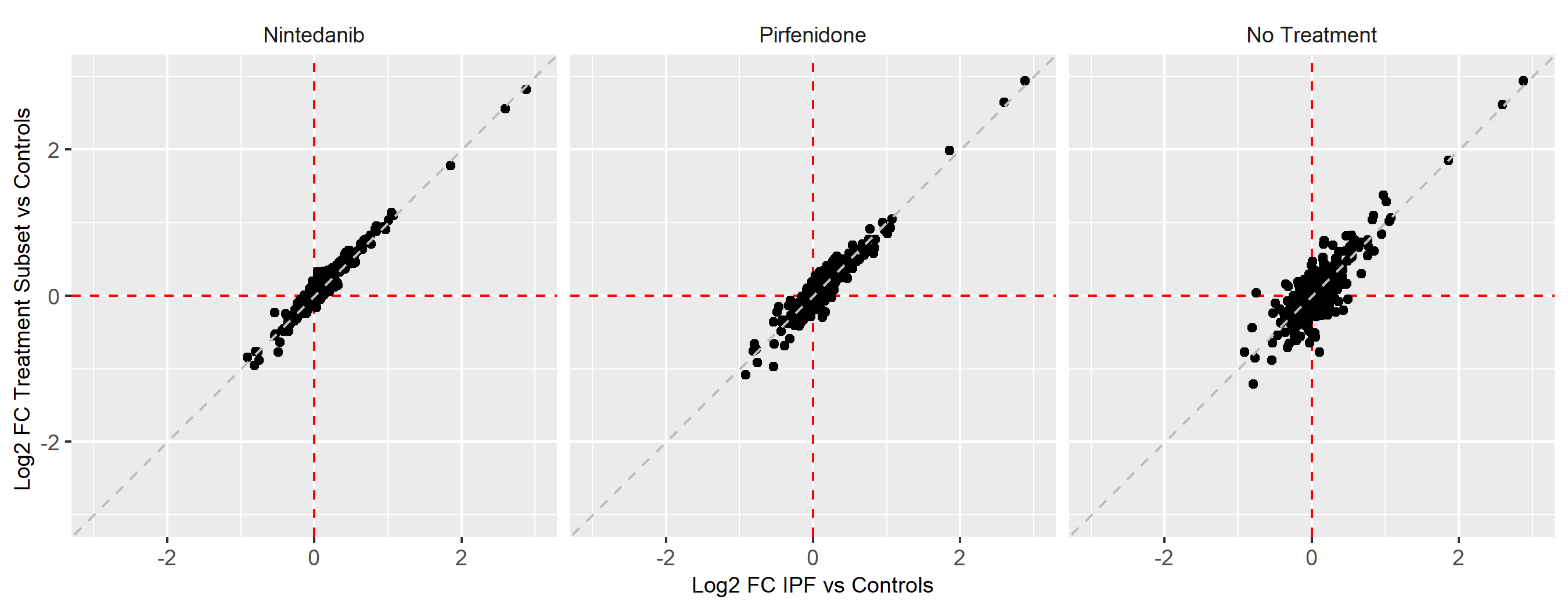


**Figure S4**. Comparisons of protein abundance between subsets of patients with IPF based on antifibrotic treatment status at enrollment. **A**) Nintedanib versus no treatment, **B**) pirfenidone versus no treatment, and **C**) nintedanib versus pirfenidone.

**A**
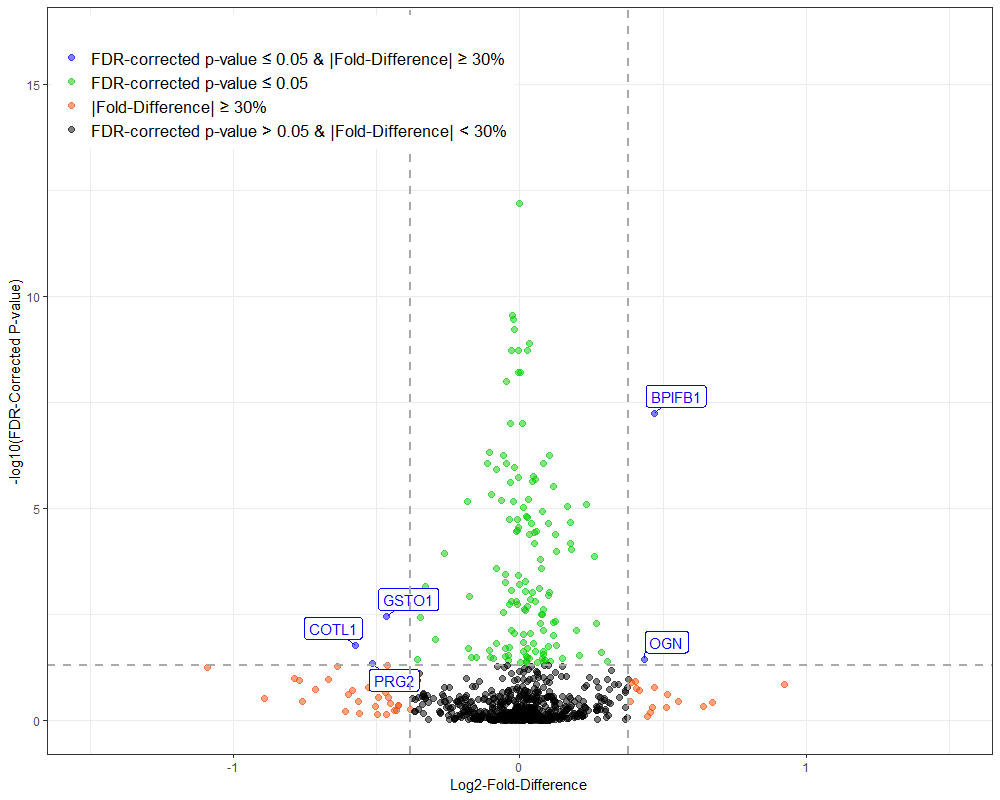


**B**
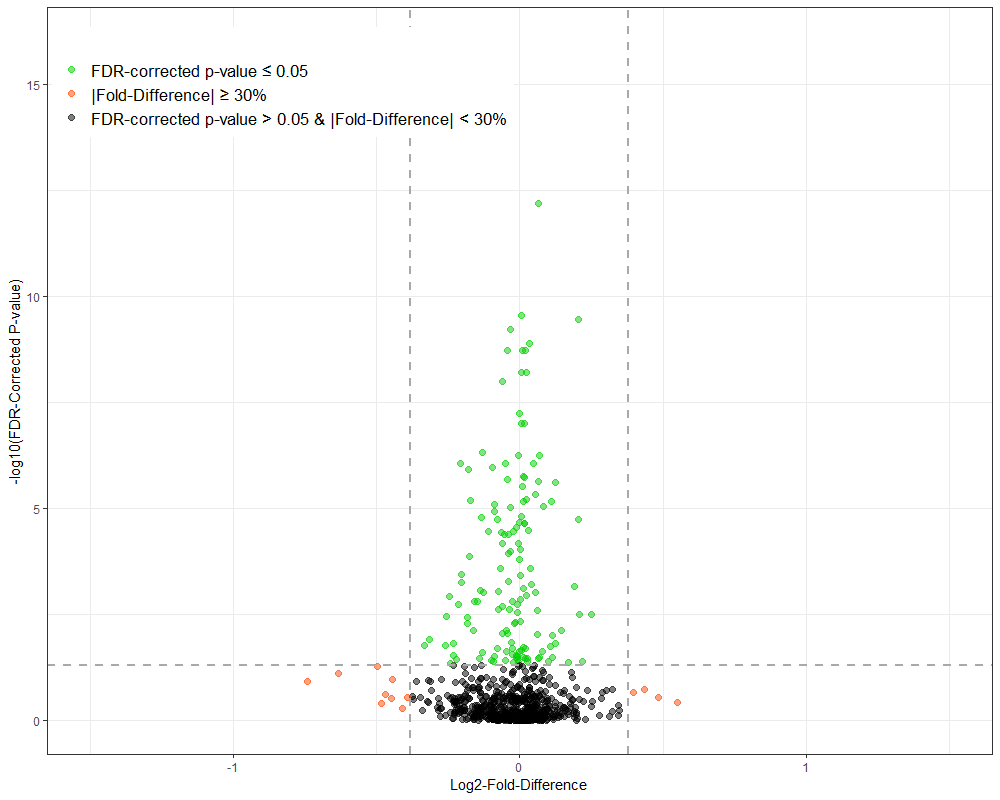


**C**
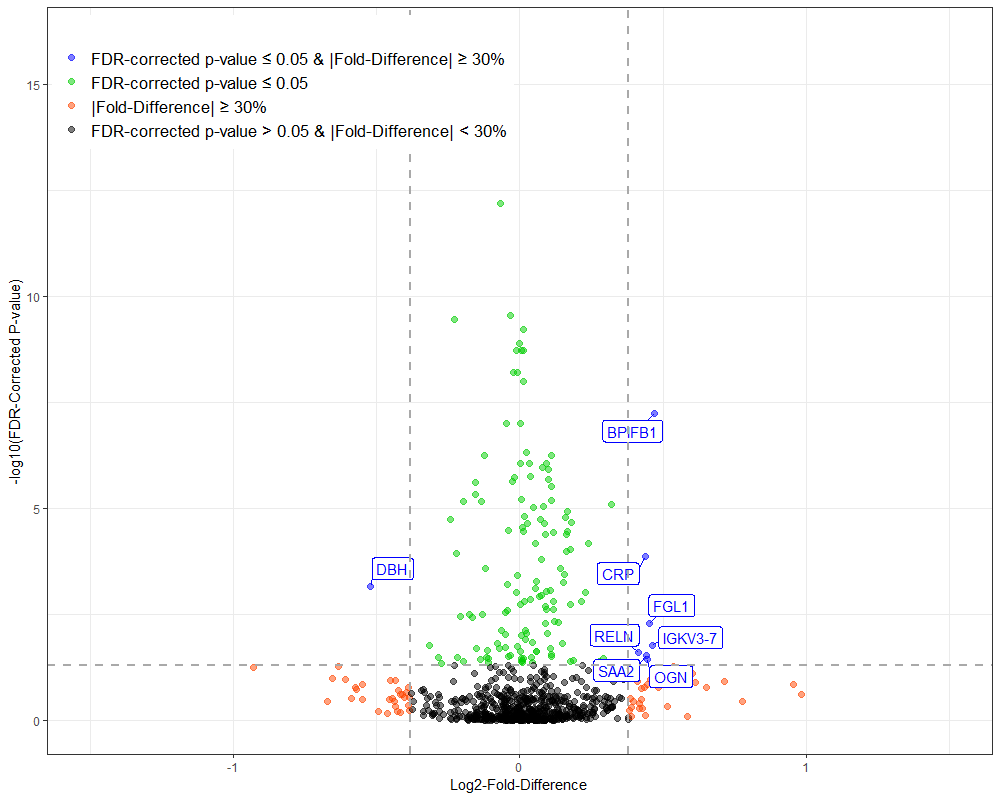


**Figure S5.** Associations between proteins and decline in FVC % predicted ≥10%, death, or lung transplant in patients with IPF in **A**) analyses unadjusted for clinical covariates and **B**) analyses adjusted for clinical covariates at enrollment.

**A**
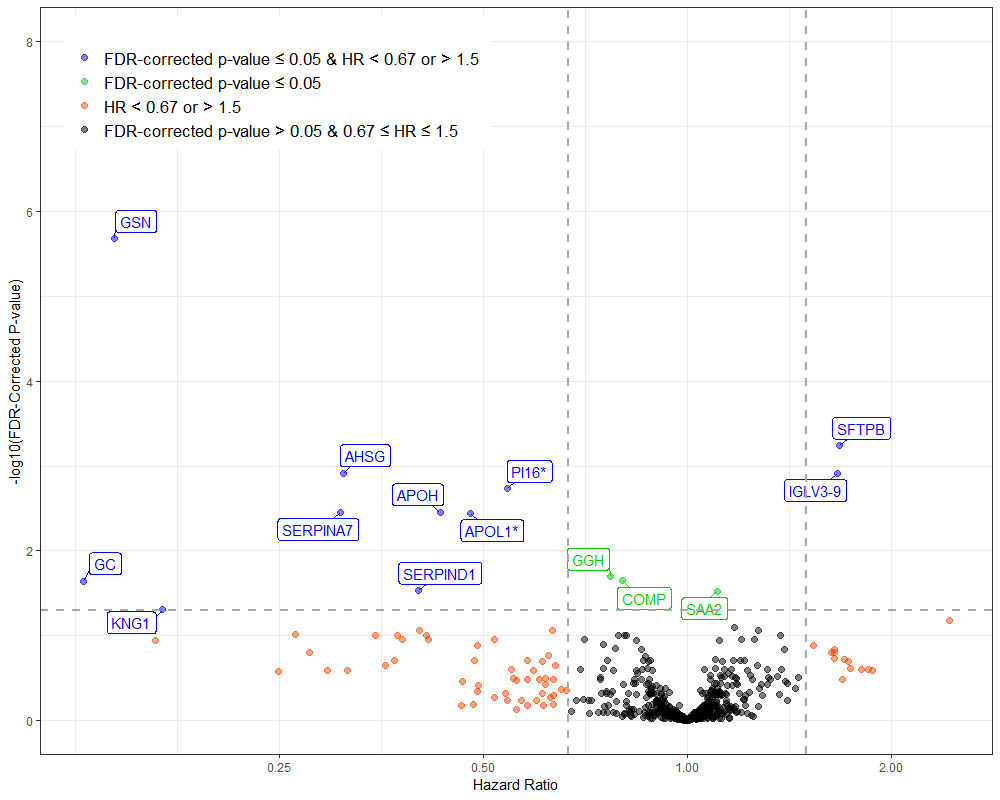


**B**
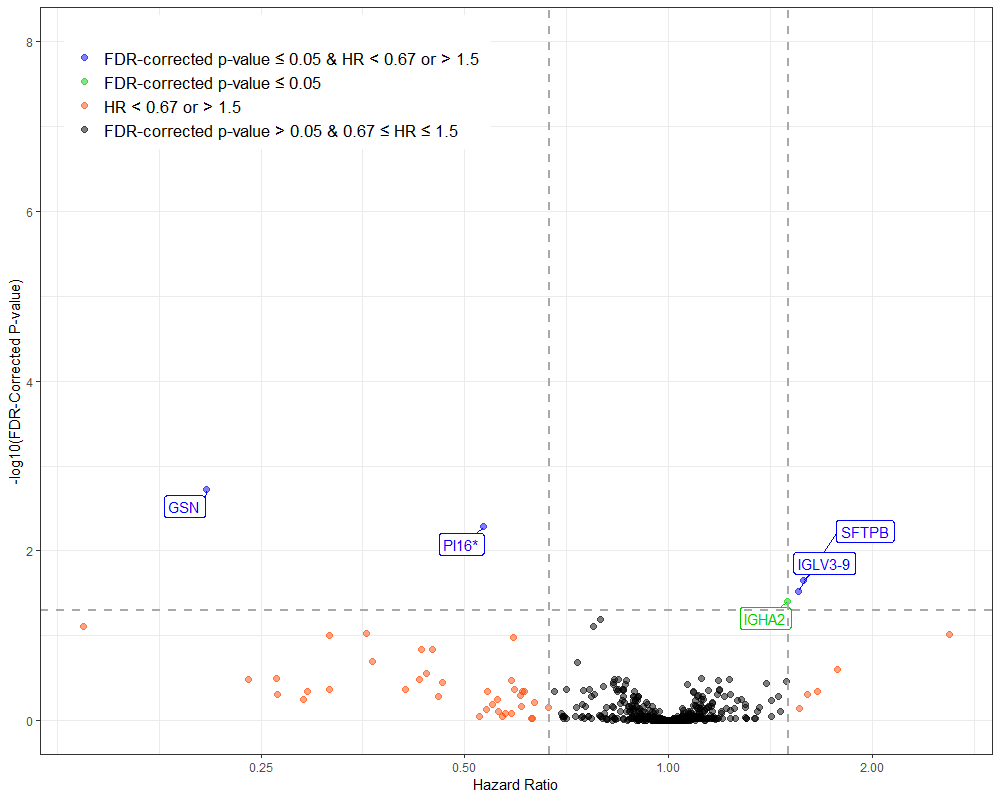


**Figure S6.** Model calibration (observed vs predicted event rates) at 6 months, 1 year, and 2 years for the composite of respiratory death and lung transplant in the training and test sets for models considering **A**) proteins only, **B**) proteins and clinical factors, and **C**) clinical factors only in patients with IPF.

**A B C**


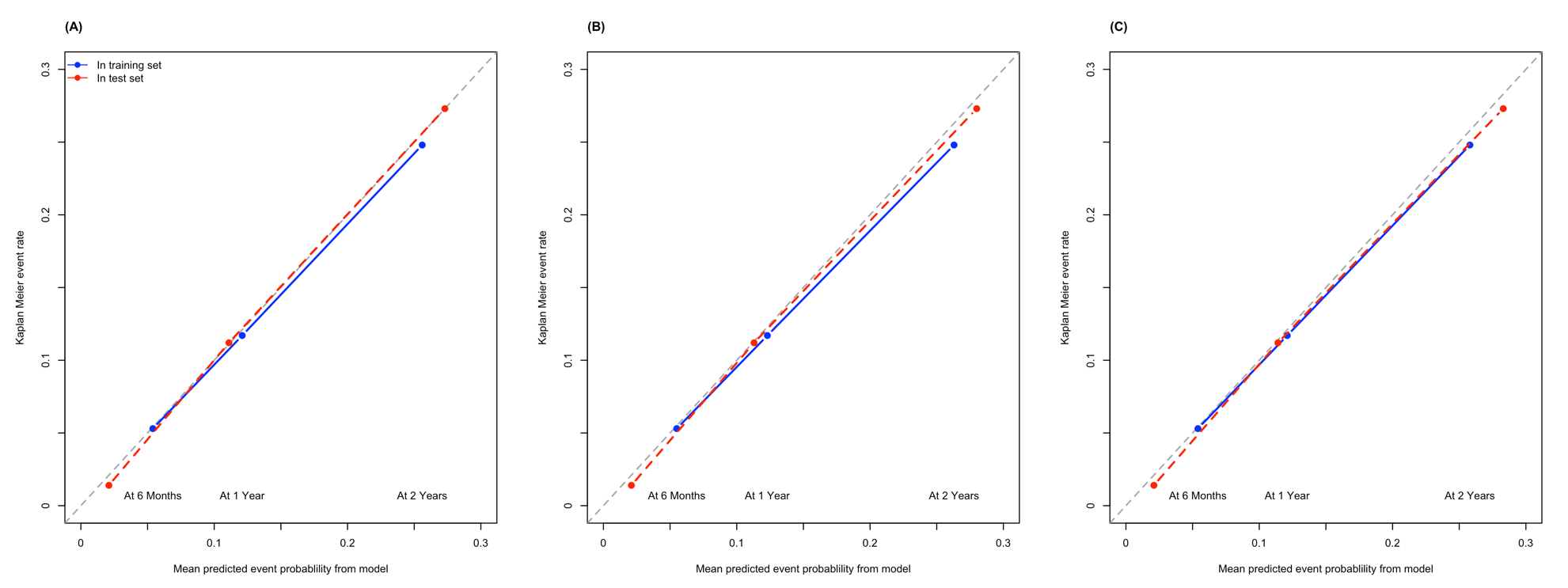

Supplement: Supplementary file 1 — Supplementary Material 1. [file 12931_2025_3377_MOESM1_ESM.docx]
